# Identification of crop cultivars with consistently high lignocellulosic sugar release requires the use of appropriate statistical design and modelling

**DOI:** 10.1186/1754-6834-6-185

**Published:** 2013-12-21

**Authors:** Helena Oakey, Reza Shafiei, Jordi Comadran, Nicola Uzrek, Brian Cullis, Leonardo D Gomez, Caragh Whitehead, Simon J McQueen-Mason, Robbie Waugh, Claire Halpin

**Affiliations:** 1 Division of Plant Sciences, College of Life Sciences, University of Dundee at The James Hutton Institute, Invergowrie, Dundee DD2 5DA, UK; 2 The James Hutton Institute, Invergowrie, Dundee DD2 5DA Scotland, UK; 3 National Institute for Applied Statistics Research Australia, University of Wollongong, Wollongong, NSW 2522, Australia; 4 Computational Informatics, Commonwealth Scientific and Industrial Research Organisation (CSIRO), Canberra, ACT 2600, Australia; 5 Biology Department, Centre for Novel Agricultural Products (CNAP), University of York, Wentworth Way, York YO10 5DD, UK

**Keywords:** Multi-phase experiment, Multi-environment trial, Saccharification, Barley, Phenotyping, Second generation biofuels

## Abstract

**Background:**

In this study, a multi-parent population of barley cultivars was grown in the field for two consecutive years and then straw saccharification (sugar release by enzymes) was subsequently analysed in the laboratory to identify the cultivars with the highest consistent sugar yield. This experiment was used to assess the benefit of accounting for both the multi-phase and multi-environment aspects of large-scale phenotyping experiments with field-grown germplasm through sound statistical design and analysis.

**Results:**

Complementary designs at both the field and laboratory phases of the experiment ensured that non-genetic sources of variation could be separated from the genetic variation of cultivars, which was the main target of the study. The field phase included biological replication and plot randomisation. The laboratory phase employed re-randomisation and technical replication of samples within a batch, with a subset of cultivars chosen as duplicates that were randomly allocated across batches. The resulting data was analysed using a linear mixed model that incorporated field and laboratory variation and a cultivar by trial interaction, and ensured that the cultivar means were more accurately represented than if the non-genetic variation was ignored. The heritability detected was more than doubled in each year of the trial by accounting for the non-genetic variation in the analysis, clearly showing the benefit of this design and approach.

**Conclusions:**

The importance of accounting for both field and laboratory variation, as well as the cultivar by trial interaction, by fitting a single statistical model (multi-environment trial, MET, model), was evidenced by the changes in list of the top 40 cultivars showing the highest sugar yields. Failure to account for this interaction resulted in only eight cultivars that were consistently in the top 40 in different years. The correspondence between the rankings of cultivars was much higher at 25 in the MET model. This approach is suited to any multi-phase and multi-environment population-based genetic experiment.

## Background

Second generation lignocellulosic biofuels can be made from biomass of dedicated energy crops (for example *Miscanthus* and willow) that are able to grow on low grade non-arable land, or from co-products of food crops such as cereal straw [1]. The efficiency of processing lignocellulosic materials to produce biofuel will depend, in part, on the accessibility of cell wall polysaccharides to enzymatic breakdown that releases fermentable sugars, a process known as saccharification. For this reason, many lignocellulosic biofuel research initiatives include large-scale screening of the saccharification properties of wide germplasm collections of crops with potential use for second generation biofuel production. Cultivars with a greater genetic capacity for releasing sugars are desirable as this would mean a reduction in production costs. Such screens typically involve large numbers of individuals with biological and technical replication, and several bespoke high-throughput saccharification assay platforms have been developed to handle large sample numbers and provide the necessary phenotypic data [2-6]. However, to date, little attention has been focused on the challenge of ensuring that the statistical design and analysis of these large and lengthy experiments are sufficiently robust to guarantee meaningful data that identifies the best performing genotypes/cultivars in a reproducible way. Achieving such rigour is essential when the phenotypic data is intended to underpin subsequent genetic dissection of the genes and loci controlling saccharification as a quantitative trait, or identifying better cultivars for biofuel applications.

The saccharification yield of any group of cultivars under testing will depend on both genetic and non-genetic factors. As the primary interest of population-based genetic studies is focused on the impact that genetic sources of variation have on sugar release, an accurate measure will only be possible if the non-genetic sources of variation can be accounted for and removed from the analysis. Such non-genetic sources may be substantial and may include variation introduced in the field during growth, during harvesting and sampling, and in the laboratory during saccharification testing. These can be quantified and accounted for by appropriate experimental design and subsequent statistical analysis.

We have been taking a genetic approach that involves screening barley straw from many hundreds of cultivars to identify genes and genotypes that have high saccharification potential. The experiment involves both field and laboratory phases. In the field, the level and composition of the lignocellulose in the straw, and therefore saccharification potential, may be affected by a range of external factors including water gradients, ultraviolet (UV)-B radiation, wind, and temperature [7,8], all of which vary in different years. The impact of these environmental factors will also vary depending on the spatial position of the cultivars in the field. Controlling for these extraneous sources of variation will ensure more accurate cultivar estimates are found [9]. Variation can also be introduced in the laboratory by different operators during sampling of harvested material, and the amount of sugar released by cultivars may depend on, among other things, differences in reagent concentration and batches, reagent contact time, heating temperatures, and gradients. Also, for a large-scale screening experiment that employs a high-throughput platform where thousands of samples are compared, processing may extend across many batches and can continue over weeks or even months. As the impact of these factors on sugar release can be sizeable, being able to control for them through appropriate design and analysis is vital.

The importance of experimental design, which generally includes replication and randomisation, in a single phase experiment (for example a field experiment) has been widely accepted since its introduction [10]. However, multi-phase experiments where the field samples are subsequently processed in the laboratory offer additional challenges. The need for laboratory duplicates, sample re-randomisation, appropriate technical replication, and batch to batch (temporal) controls combined in an appropriate analysis is often overlooked. Furthermore, complementary designs at both phases ensure that field and laboratory variation can be separated and accounted for correctly in the analysis.

The literature to support the use of appropriate multiple-phase design and analysis has grown slowly over the last 50 years. McIntryre and others [11-15] discuss the analysis of multi-phase experiments in general. Indeed, some progress has been made, particularly relating to the benefits of a multi-phase design and analysis in crops [16-19]. In particular, Butler *et al*. [17] show the existence of substantial non-genetic sources of variation in field and laboratory and the need to account for these to improve the accuracy of the phenotyping, concluding 'the use of a multi-phase design and analysis is superior to an approach which doesn’t use a valid statistical design and efficient analysis’. In the case of large-scale saccharification screens, although many of the technical challenges have been identified [20], most studies have not exploited the opportunities offered by appropriate statistical design in extracting meaningful data while controlling for such technical 'noise’. In the laboratory phase in particular, while sample replication is included in most studies, the value of both spatial and temporal randomisation is usually overlooked. By ignoring many non-genetic sources of variation, and simply averaging over biological and technical replicates to obtain a cultivar value, cultivar selection may not be optimal and any planned subsequent genetic studies such as quantitative trait loci (QTL) mapping may be compromised.

For crop plants, cultivar performance is affected by the environment, such as different growing times (for example season or year) or locations. To assess this cultivar by environment interaction, and thus the stability and adaptability of cultivars, a multi-environment trial (MET) can be undertaken in which the experiment is wholly repeated in different locations. Smith *et al*. [21] provides a comprehensive review of mixed model approaches to the analysis of METs, which considers the phenotypic response as partitioned into cultivar effects, environmental effects, cultivar by environment interaction effects, and within-environment effects. For a multi-phase experiment the within-environmental effects of a MET will include the non-genetic field and laboratory variation found in each environment.

The study described here illustrates the practical benefit of accounting for both multiple phases and multiple environments through sound experimental design and analysis. We grew 648 and 856 elite cultivars of spring barley in an experiment consisting of field and laboratory phases conducted and repeated over two years (referred to as 'trials’). Our aims were to characterise and rank existing elite cultivars for their possible use as parents in barley improvement programmes to improve the saccharification potential of the straw, and to subsequently use the phenotypic values to accurately identify regions of the barley genome (genes or loci) associated with high straw saccharification potential.

## Results and discussion

**Figure 1:**
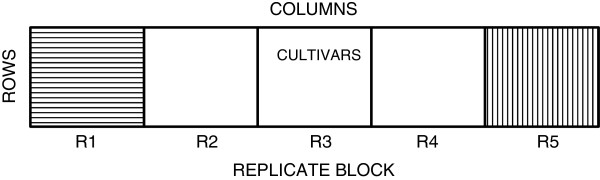
**The spatial row-column field design for the 2010 trial.** In 2010, 648 barley cultivars were grown in five replicate blocks, R1 to R5, with cultivars randomised within a block. In 2011, 886 cultivars were grown in the same way. A vertical stripe in R5 highlights field column direction and the horizontal stripe in R1 highlights field row direction.

In each trial, the barley cultivars were grown in pots in the field in a polyethylene tunnel arranged in a spatial row-column design with five replicate blocks (Figure 1). There were 648 cultivars planted in each replicate block in the 2010 trial and 856 cultivars planted in each replicate block in the 2011 trial, with 639 cultivars common to both trial years. This resulted in a total of 3,300 and 4,480 harvested straw samples from the 2010 and 2011 trials, respectively, which included five true biological replicates per cultivar (Table 1). The straw samples were milled to a powder.

**Table 1:** Details of the laboratory design

**Trial**	**Number of cultivars processed**^**a**^	**Number of samples per replicate block**^**b**^	**Number of samples**	**Plates**
2010	647	660	3,300	165
2011	856	900^c^	4,480	224

^a^Total number of cultivars across all five replicate blocks; ^b^includes duplicated cultivars used as laboratory replicates; ^c^replicate 1 had 880 samples.

**Figure 2:**
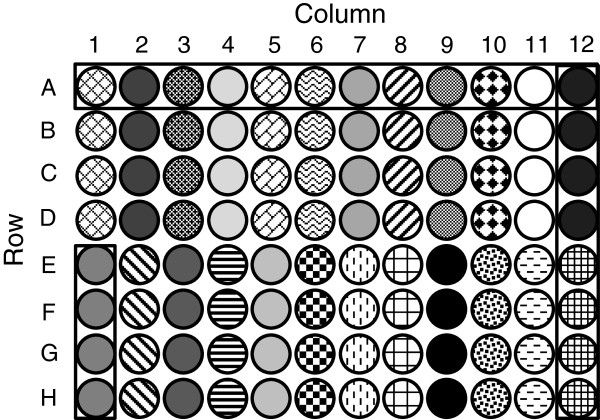
**Sample layout within each 96-well plate.** Each sample has four technical replicates and is shown in a different shade or pattern: columns 11 and 12 contain standard samples, and the rest of the samples are test cultivars. The wells of plate row A (wells A1 to A12) and plate column 12 (wells A12 to H12) are boxed as well as one of the 24-plate plots (wells E1 to H1).

**Figure 3:**
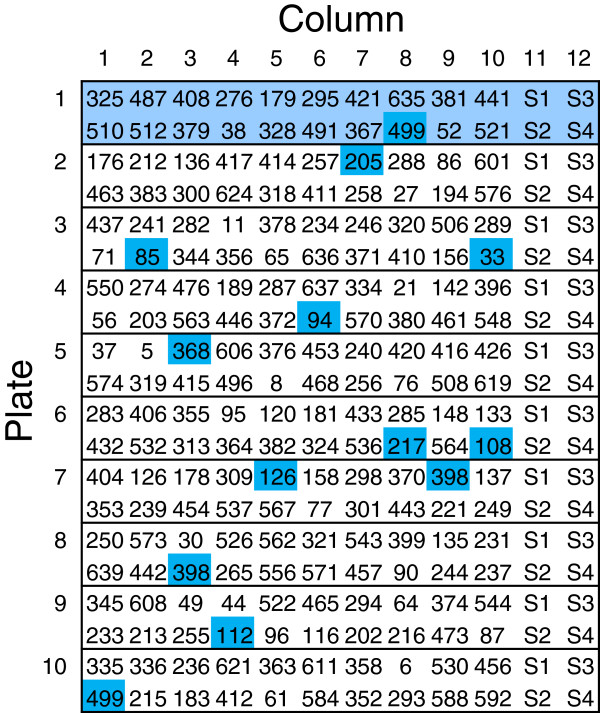
**Subset of the laboratory design of replicate block, showing the sample allocation and duplicated cultivars.** The subset is the first ten plates of replicate block R1 (2010 trial). The cultivar numbers are shown; highlighted in dark blue are laboratory duplicated cultivars, and Plate 1 has been highlighted in light blue. Each cultivar number represents four replicate samples as illustrated in Figure 2. Columns 11 and 12 contain the same standard samples on each plate.

**Figure 4:**
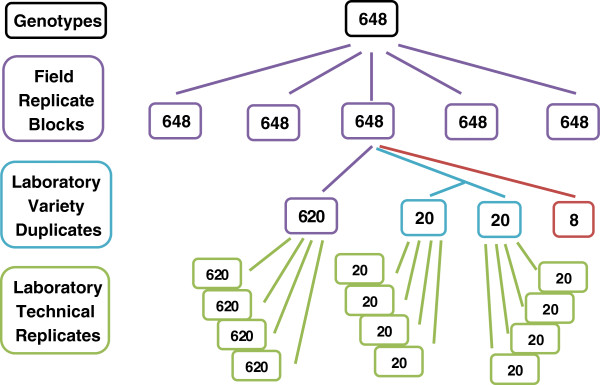
**Summary of the field and laboratory design phases, following through a single field replicate block**. Laboratory processing of all field replicate blocks in both trials was undertaken; however, the follow-through for only one of the replicates is shown, which is replicate block R1 (2010 trial). There were eight missing cultivars in this particular replicate (shown in red). Overall, 660 (620 + 20 + 20) samples were processed in the laboratory, representing 640 unique cultivars out of 648 total cultivars with 20 of these 640 cultivars duplicated. A similar diagram is applicable for the 2011 trial.

For each replicate block, in each trial, the milled straw sample from each available cultivar was re-randomised to a 96-well plate. Each plate contained four technical replicates of each sample (Figure 2). In addition to the re-randomisation and technical replication of samples, a subset of cultivars were chosen as duplicates and randomly allocated across the plates using a partially replicated design (Figure 3). This additional level of replication enabled us to determine laboratory variation across Plates. A summary of the field and laboratory design phases for trial 2010 is shown in Figure 4.

The assay for sugar release performed on the 96-well plates resulted in 13,200 and 22,480 data points (consisting of the results of four technical replicates plus replicated cultivars) in 2010 and 2011, respectively. Laboratory processing of the 165 and 224 plates (Table 1) took 8 and 15 weeks for the 2010 and 2011 trials, respectively. For each data point, details from the field (row, column, and replicate block), laboratory (day of processing, plate, plate row, and plate column), and the trial (2010 and 2011) were recorded for subsequent data analysis.

**Figure 5:**
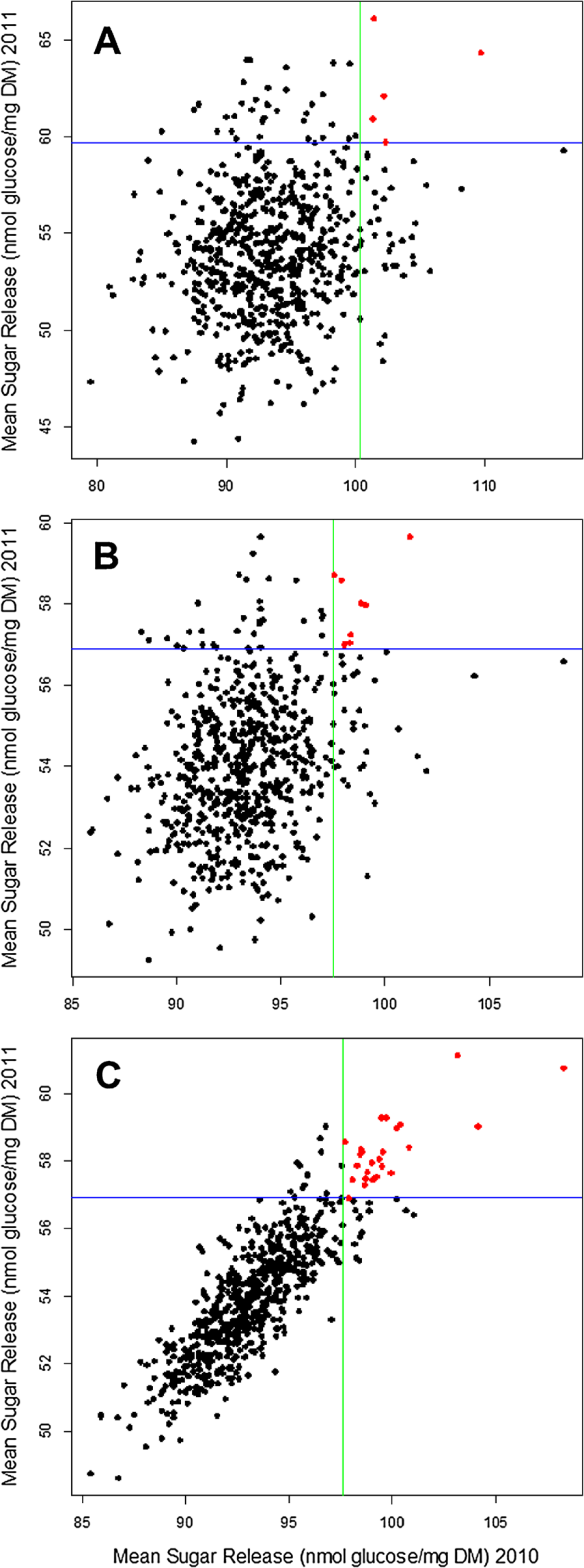
**Sugar release of each cultivar in 2010 and 2011 trials using models A, B and C. (A)** The unadjusted mean sugar release (nmol glucose/mg DM) of each cultivar in each trial. **(B)** The cultivar mean sugar release after adjustment for field and laboratory variation, single trial analyses. **(C)** The cultivar mean sugar release after adjustment for field and laboratory variation, MET analysis. A, B, and C correspond to the output from models A, B, and C, respectively (Table 2). Cultivars to the right of the green line are the top 40 cultivars for sugar release in 2010. Cultivars above the blue line are the top 40 cultivars for sugar release in 2011. Cultivars highlighted in red are the cultivars that are consistently in the top 40 for sugar release in both years 2010 and 2011. There were five cultivars in the top 40 in both years for model A, eight cultivars for model B, and 25 cultivars for model C. DM, dry matter; MET, multi-environment trial.

The data was analysed according to three distinct statistical models (see Table 2 for summary and Methods section in conjunction with Tables 3 and 4 for a full description of the models). Model A was a baseline model that did not account for the field, laboratory, or environment variation. This model represents the results obtained if all the observations from a particular cultivar are simply averaged and reflects the common practice in most high-throughput saccharification studies to date. Model B took the variation introduced in the field and laboratory into account and adjusted for both, but, like model A, did not consider the multi-environment variation of growing the plants in two successive years (that is, it was equivalent to fitting each trial separately). Only model C took all sources of variation into account, adjusting for field, laboratory, and the multi-environment variation between the trials in 2010 and 2011. Figure 5A shows the results of the sugar release of each cultivar for both trials and is the result of model A, Table 2. The level of sugar release was lower in 2011 with an overall mean of 54.0 nmol glucose/mg DM (dry matter) as compared to an overall mean of nearly double that of 93.3 nmol glucose/mg DM in 2010. This illustrates the scale of variability that the environment (growth in one or other trial year) and/or the laboratory (analysis in one or other trial year) can introduce into the data. Irrespective of this, the lack of any pattern in the data in Figure 5A suggests that systematic differences alone cannot explain the very low correlation between the raw mean sugar release of cultivars across the two trials. Significant levels of random variation must also influence the results unless it is assumed that genetic differences between cultivars truly exert no consistent influence on saccharification properties, a premise that is unlikely at best.

**Table 2:** Summary of the models fitted and their corresponding log likelihood

**Model**	**Equation**	**Field and laboratory terms**^**a**^	**Cultivar by trial interaction**^**b**^	**Log likelihood**	**Number of variance parameters**	**Heritability**	
						**2010**	**2011**
A	2	***Z***_*u*_ ***u*** = 0	$\theta_{g_{1}g_{2}}=0$	-88376.08	4	0.12	0.20
B	1	***Z***_*u*_ ***u*** ≠ 0	$\theta_{g_{1}g_{2}}=0$	-70884.82	26	0.37	0.43
C	1	***Z***_*u*_ ***u*** ≠ 0	$\theta_{g_{1}g_{2}}=0.66$	-70853.58	27	0.46	0.49

^a^ ***Z***_*u*_ ***u*** = 0 implies the terms indexed with a L or F are not included in the model (see Methods and associated Tables 3 and 4); ^b^$\theta_{g_{1}g_{2}}=0$ implies the genetic covariance between the two trials is zero and that no cultivar by trial interaction is fitted. This is equivalent to fitting the two trials separately.

**Table 3:** Tiers for the factors of non-genetic sources of information from the field and laboratory phases

**Tier 3**	**Tier 2**	**Tier 1**
**Factors randomised in field phase**	**Factors unrandomised in field phase**	**Factors unrandomised in laboratory phase**
Cultivar	Field block	Day
	Field row	Plate
	Field column	Plate plot^a^
		Plate row
		Plate column

^a^Plate plot refers collectively to the four consecutive wells used for a single sample. The aim of this stage in the analysis process is to identify the objects involved in the randomisation and then to determine the factors associated with each set of objects; these groups of factors are referred to as tiers. Here the factors form the non-genetic sources of information from the experimental design elements of the field and laboratory phases. Following Brien [12] the tiers are labelled in reverse chronological order of conduct of the experiment.

**Table 4:** ANOVA table showing sources of variation from the field and laboratory and corresponding model terms

**Source of variation**	**Model term**
**Laboratory plate**	
Field block	F block
Residual	L day
	L day x L plate
**L plate x Laboratory plate plot**	
Field plot	
*Cultivar*	Cultivar
*Residual*	F row x F column
	F row
	F column
Residual	L plate x L plate plot
	L plate x L column
	L plate x L row
	L row
	L column
	L row x L column
**L plate x L plate plot x Laboratory technical replicates**	Residual

For the design factors shown: L, laboratory; F, field; x, represents an interaction between terms; L plate, refers to the 96-well plate used in the laboratory phase; L row and L column, refer to rows and columns of the plate; and L plate plot, refers collectively to the four wells that contain the technical replicates of a single sample.

In order to attempt to extract the non-genetic sources of variation, the data was analysed according to two additional models (Table 2) that illustrate the benefits of accounting for field and laboratory variation (that is, the multi-phase nature of each trial) and cultivar by trial interactions (that is, the multi-environmental nature of the trials). It should be noted that the ability to partition the variation into field and laboratory strata in the models is the result of the sound statistical design employed. While model A ignores these sources of variation (see Methods section; terms indexed with an L or F in Table 4 are excluded), model B accounts for the field and laboratory variation (see Methods; terms indexed with an L or F in Table 4 are included). In both models A and B, the analysis of each trial is conducted separately, so that no cultivar by trial interaction is fitted. The third model (model C) includes both the field and laboratory variation and a cultivar by trial interaction. The influence that model B and particularly model C have in refining the data can be clearly seen in Figure 5. While model B (Figure 5B) reduces the spread of the data compared to model A (Figure 5A), a strong, broadly linear correlation between the two years’ data is only clearly apparent in the output from model C (Figure 5C). The overall correlation between the two years’ data represented in Figure 5C is 0.66 or 66% (see Table 2).

A log likelihood ratio test is used to determine which model fits best (Table 2). Model C, the full model which accounts for field and laboratory variation and includes a cultivar by trial interaction, is the best fitting model and significantly better (*P* < 0.001) than model B. Similarly, model B, which accounts for field and laboratory variation, is significantly better (*P* < 0.001) than model A.

The precision of cultivar effects under the three models can be determined by examining the average prediction error variance, which is a measure of the difference between the true and predicted cultivar effects. For models A, B, and C this was 2.61, 2.44, and 2.28, respectively, suggesting that model C is the one that most accurately represents the cultivar effects. Heritability provides an indication of the amount of variation in sugar release that is due to the genetic variation among cultivars and can be calculated separately for both trials for all models (see Methods section). Using model A, the heritability in the 2010 trial is just 0.12 (that is, only 12% of the variation is due to genetics), while that of the 2011 trial is 0.20 (Table 2). The benefit of including field and laboratory variation in the model (model B versus A, Table 2) is apparent as there is a substantial increase in heritability in both trials. Nevertheless, had the trial only been performed in 2010, the heritability after adjusting for field and laboratory variation, would not have risen above 0.37 (37%). Including a second year of trial in 2011 and accounting for the cultivar by trial interaction (model C versus B, Table 2) achieves a further significant improvement in the proportion of the variation that can be assigned to the cultivars (heritability). By extracting non-genetic sources of variation (from field, laboratory, and trial/environment) the heritability rises to 0.46 for the 2010 trial and 0.49 for the 2011 trial, that is almost 50% of the variation being considered is now due to genetics when the best model, model C, is used. The estimated variance components for model C (Table 2) are examined in Table 5. Both the field and laboratory made a substantial contribution to the total non-genetic variation present within each trial. The field variation was 27.9% and 22.2% and the laboratory variation was 56.7% and 58.1% of the total variation in the 2010 and 2011 trials, respectively (Table 5). This concurs with the findings of Smith *et al*. [18] who found that variation in the laboratory accounted for the greatest proportion (58%) of the non-genetic variation in a study where field wheat trials were followed by laboratory grain milling to estimate flour yields. This highlights the importance of accounting for the laboratory phase and not just the field phase in the statistical model; without this, half or more of the non-genetic variation is not accounted for leading to unnecessary 'noise’ in the data and potentially confounding valid conclusions being drawn.

**Table 5:** Summary of the REML estimates of variance components and the corresponding percent of total variation

**Variance component**^**a**^	**2010 trial**	**Percent (%)**	**2011 trial**	**Percent (%)**
**Field**				
F block	54.0	20.5	0.7	0.7
F column	2.1	0.8	0.8	0.9
F row	0.4	0.2		
F row x F column	16.8	6.4	19.1	20.6
**Field Total**	73.3	27.9	20.6	22.2
**Laboratory**				
L day	32.2	12.2	1.6	1.8
L day x L plate	28.3	10.8	10.0	10.7
L plate x L plot	37.5	14.2	18.3	19.6
L column	4.1	1.6	4.8	5.1
L row	12.8	4.9	8.5	9.1
L plate x L column			2.7	3.0
L plate x L row	32.5	12.4	7.4	8.0
L row x L column	1.6	0.6	0.7	0.8
**Laboratory Total**	149.0	56.7	54.0	58.1
**Cultivar**	13.5	5.1	6.9	7.4
**Residual**	27.4	10.4	11.5	12.3

^a^Variance components for sugar release using model C, Table 2. REML, residual maximum likelihood; x, represents an interaction between terms.

In our experiment, some of the sources of variation had recorded events associated with them. For example, the largest percent variation in the field component in the 2010 trial was block (Table 5). A failure in the automatic water system was quickly rectified by supplementary watering but a small area of plants suffered a short period of drought stress and flowered earlier than the others. The subsequent effect on saccharification is captured in the field blocks, hence the high percent variation. For the laboratory variance components, some of the 12.2% variation due to day of processing in the 2010 trial was attributed to the saccharification reagents being freshly prepared every few days. For this reason, in the 2011 trial, one large reagent batch was prepared at the beginning of the analysis and used for processing of all the plates resulting in a much lower total variation due to day of processing of 1.8%. Clearly, unexpected events or unanticipated factors can influence any large experiment. Appropriate modelling and analysis of the data may allow practical changes to be made to improve experimental procedures. For this particular dataset, however, through sound statistical design and analysis, the cultivar effects after adjustment will not include the variation due to these factors and the results of the experiment are therefore not compromised.

Of interest is cultivar performance and in particular the selection of cultivars that may have the best sugar release properties. Therefore, the ranking of the sugar release of the top 40 cultivars (that is, the mean for each cultivar) was used as an indicator to investigate how the results change as field and laboratory variation and, finally, the cultivar by trial interaction, are accounted for by the different models. The top 40 were chosen as this is a reasonable number of cultivars to subsequently take forward to evaluate for bioethanol production and because this number covered many of the cultivars that looked like outliers having substantially better sugar release than most of the cultivars within the population in each year (that is, cultivars above the blue line and to the right of the green line in Figure 5A).

The effect of accounting for field and laboratory within each trial was investigated, by comparing where each cultivar would be placed if cultivars were ranked by mean sugar release using model B (Table 2) that adjusts for field and laboratory variation, compared to the unadjusted model A (Table 2) where no terms relating to field or laboratory variation are included. Both models assume no cultivar by trial interaction (the genetic covariance $\theta_{g_{1}g_{2}}$ is zero), which is equivalent to fitting a separate model for each trial. For the 2010 trial this comparison reveals that only 26 of the cultivars in the top 40 are the same for model A versus model B. This suggests that 14 (35%) of the cultivars that appear in the top 40 in model A are there only because errors introduced in the field and the laboratory have not been accounted for. Conversely, 14 cultivars that reach the top 40 when field and laboratory variation is extracted from the data (model B) are not identified as being in the top 40 by model A. Clearly these discrepancies would have a serious impact on the appropriateness of the cultivars selected for further investigation if only model A was used, or, indeed, if the trial had only been performed in a single year, in 2010. For the 2011 trial, the number in agreement is higher at 32; however, there is still a discrepancy between the models with eight cultivars different between the models A and B.

The importance of including all of the data in a single model (model C, Table 2) that is multi-phase (adjusting for field and laboratory variation) and multi-environment (cultivar by trial interaction) is that comparisons between trials can now be made. This is the comparison that is most relevant if cultivars that consistently show high sugar release in multiple years and environments are to be selected. As a base-line for the comparison, consider the mean sugar release of cultivars that consistently appear in the top 40 in both trials using model A (Table 2), which did not adjust for field and laboratory variation nor consider a cultivar by trial interaction. Model A identifies only five cultivars (SCRI_S_0010860, Baudin, SCRI_S_0006956, Mandolin-1418, Aapo) as being in the top 40 ranking cultivars in both trials (highlighted in red in Figure 5A).

The effect of accounting for the cultivar by trial interaction on mean sugar release of cultivars can be considered by comparing the results of model B and C (Table 2). Both models account for field and laboratory variation but model B assumes there is no cultivar by trial interaction (the genetic covariance between the two trials $\theta_{g_{1}g_{2}}$ is zero), whereas model C (Table 2) includes a cultivar by trial interaction (the genetic covariance between the two trials $\theta_{g_{1}g_{2}}$ is non-zero). Figure 5B shows that application of model B results in only eight cultivars (SCRI_S_0010860, SCRI_S_0006956, Bulbul 89, Keops, Gant, Aapo, Mandolin-1418, Fontana) that consistently rank in the top 40 across the two trials. By comparison, Figure 5C shows the results of model C (that accounts for a cultivar by trial interaction), which identifies 25 cultivars as ranking consistently in the top 40 in both trial years. Despite the variation between the trials in the amounts of sugar released by each year of analysis, this model maintains a strong genetic correlation between the trials of 0.66. Because of the stability of their ranking positions across two years, these cultivars present the most suitable candidates for further investigation. These cultivars are Sterling, Hamelin, SCRI_S_0010860, Baudin, SCRI_S_0006956, Bulbul 89, Spiral, Mandolin-1418, Keops, Dew, Fontana, Gant, Annabell, Fairytale, Aapo, Juno, Safir, 915006, Romi, SW 2808, Tokak, Skiff, Deba, Abed Otis, and SJ Christina. Interestingly, many of these are early maturing cultivars with heading dates (that is, time to maturity) of less than 70 days.

## Conclusions

Our analyses illustrate the benefit of accounting for both the multi-phase and multi-environment aspects of an experiment in which we aimed to classify and rank barley cultivars according to the saccharification potential of their straw.

A sound statistical design was produced for both phases of the experiment for each trial. In the field phase, five biological replicates of each cultivar were grown in a spatial row-column arrangement. A partially replicated design that re-randomised samples from the field, duplicated between 3% and 5% of cultivars, and included technical replicates, was used for the laboratory phase that tested for sugar release.

The approach to analysis took account of design factors in the field and laboratory. Trends due to spatial position in the field described by replicate block, field columns, and rows were accounted for in the analysis and were shown to represent between 20% and 30% of the total variation in sugar release. Laboratory factors, such as day of processing, plate, and position of samples within plates, were also accounted for in the analysis and were found to contribute between 50% and 60% of the variation in the sugar release. Most high-throughput saccharification studies published to date have simply averaged over biological and technical replicates to obtain a cultivar value and have disregarded the impact of other potential sources of non-genetic variation such as time and date of analysis and position of samples within incubators. Lack of consideration of these kinds of variables can invalidate subsequent research, for example, see Smith *et al*. [22] where consecutive rather than randomised milling of replicate samples led to detection of a false QTL that was simply a consequence of mill day. We have shown here that, for high-throughput saccharification analyses, the partitioning of the variation in sugar release to include these non-genetic sources ensures that the cultivar means are more accurately represented than if the non-genetic variation was ignored, and decisions on cultivar selection (or subsequent genetic analyses) can consequently be made with more certainty. In addition, the heritability was more than doubled in 2010 and 2011 by including the field and laboratory variation in the analysis, illustrating the benefit of this approach.

Cultivar performance was noticeably affected by trial year with much lower sugar release being achieved in 2011. The importance of including a cultivar by trial interaction and therefore fitting a single model that in addition to the field and laboratory factors includes a multi-environment aspect was revealed by the changes in the cultivars present in the top 40. Failure to account for the interaction resulted in only eight cultivars that were consistently in the top 40, whereas the correspondence between the ranking of cultivars was much higher at 25 using the MET model. In this dataset there were just two trials and therefore the structure of the genetic variance matrix for trials was straight-forward, allowing for a single covariance component. Multi-environment experiments with more than two trials can be harder to fit, particularly if the genetic variance matrix for trials is fitted as unstructured. It is recommended for METs with more than two sites that a factor analytic structure for the genetic variance matrix for trials [21] be considered. For a large number of sites, a two-stage analysis where cultivar means for each trial are obtained from the appropriate model (that includes field and laboratory components) and then used in a weighted multi-site analysis, may be necessary due to computational restrictions.

In addition to illustrating the benefits of sound statistical design and analysis, the key biological outcome of this large-scale experiment was the clear oversubscription of lines that are early maturing in the top 40 saccharifying lines. Lignin concentration in the stem increases with increased maturity [23,24] and early maturing cultivars by definition have less time for stem lignification. Reduced lignification may therefore be the single major factor that allows sugars to be released more easily from barley straw. The increased sugar release properties of early maturing cultivars may be a previously unrecognised quality characteristic that could potentially be exploited in short season environments by tailoring crop cultivars for use as both food and fuel.

In conclusion, our results show that conducting a multi-phase experiment with a sound statistical design at both phases of the experiment and analysing results appropriately improved the accuracy of cultivar means, increased heritability, and improved the coincidence between cultivars across years, ensuring that the cultivars with the best consistent sugar release are correctly identified for further investigation.

## Methods

### Barley cultivars

A total of 648 and 856 spring two-row barley cultivars were assembled to establish a multi-parent population that was suitable for ranking saccharification potential and for conducting genome-wide association studies (GWASs). The genotypes and their derivation have been described previously [25,26].

### Statistical design

#### Field

During two consecutive years, 2010 and 2011 (referred to hereafter as the 2010 and 2011 trials), spring barley cultivars were grown in 25 cm pots placed on felt matting in the field within a polythene tunnel, with each pot containing one plant (cultivar). Plants were watered daily throughout the growing season from below by irrigating the matting and received no fertiliser in addition to that included in the starting compost. They were treated with fungicide once during the growing season to control foliar pathogens (largely mildew).

In each trial, the pots were arranged in a spatial row-column design with five replicate blocks (R1, R2, R3, R4, and R5), where the replicate blocks correspond to biological replicates. In the 2010 trial, there were 648 cultivars planted with pots arranged in 405 columns by 8 rows with each replicate block consisting of 81 columns by 8 rows. In the 2011 trial, there were 856 cultivars planted with pots arranged in 535 columns by 8 rows with each replicate block containing 107 columns (Figure 1). There were 639 cultivars common to both trial years. The software CycDesigN 4.0 (VSN International Ltd, Hemel Hempstead, UK) was used to generate the design each trial year.

#### Laboratory

To quantify sugar release or saccharification, the main tiller from each plant was identified and the second internode was harvested. The internodes were dried in an oven with hot air circulation at 45°C overnight. Samples were then chopped into 2 mm wide pieces using multi-blade shredder scissors. The chopped materials were placed into 2 mL screw cap tubes and filled to the 1.5 mL level indicator which provided approximately 1.5 mm^3^ feedstock. Subsequently, two 5 mm metal beads were added to each tube. Samples were milled using a TissueLyser II (Qiagen, Limburg, Netherlands) for 1 minute 20 seconds, and rotated to ensure uniform grinding.

The milled samples were allocated to a 96-well plate (with 8 rows by 12 columns) using a grinding and loading robot (Labman Automation Ltd, Stokesley, UK), with each well containing 4 mg of milled sample. A total of 24 samples per plate were tested each with four technical replicates. Each sample was placed in four consecutive wells down the plate columns so there were two samples per column. Of the 24 samples, the cultivar samples represented 20 of these, the other four were standard samples with known sugar concentration and were confined to columns 11 and 12 (Figure 2). Between three and four plates (60 to 80 cultivar samples) were processed per day.

The pre-treatment, hydrolysis, and sugar determination of samples was performed using an automated liquid handling station (Tecan, Maennedorf, Switzerland). Each well was pre-treated with 0.5 N NaOH at 90°C for 30 minutes, after which the biomass was rinsed six times with 500 uL sodium acetate buffer. The samples were incubated while shaking at 50°C for 8 hours in the presence of an enzyme cocktail (4:1 ratio of Celluclast and Novozyme 188; Novozymes, Bagsvaerd, Denmark). Enzyme loading was approximately 6 FPU/g of material, and automated reducing sugar determination was carried out using a modification of the 3-methyl-2-benzothiazolinone hydrazone (MBTH) method, as previously described [3].

For the laboratory design, milled straw samples from the polythene tunnel experiment were re-randomised for laboratory processing. In addition, 3% to 5% of cultivars were duplicated in each trial year. The duplicated cultivars act as control cultivars and provide an indication of laboratory variation across plates (not available from the four technical replicates which were within a single plate).

This approach results in better estimates of genetic effects [27] than including samples of a single control cultivar across the plates as all resources are concentrated on cultivars of interest and additional information is gained on these duplicated cultivars.

The re-randomisation of cultivars from the field to the laboratory maintained the replicate blocks. For each trial, within each replicate block, samples from the field were re-randomised to plates using a partially replicated design [27] generated by the R package DiGGer [28]. This design ensured that the field position of samples was not confounded with the order of laboratory processing.

For each replicate block of the 2010 trial, 660 samples were processed in 33 plates, where three to four plates were processed each day. The 660 samples included cultivars available from the polythene tunnel plus a random selection of these cultivars for laboratory duplicates. The design ensures that within any one of the 33 plates which collectively encompass a replicate block, between one and two of the laboratory duplicated cultivars were included. For example, in replicate R1, 20 cultivars were duplicated, ensuring there were 40 duplicated samples across the 33 Plates. A subset of the laboratory design for replicate R1 of the 2010 trial is shown in Figure 3.

The design for the 2011 trial was similar, 900 samples were processed in 45 plates in each replicate apart from replicate R1 which, owing to the number of missing cultivar samples from the field, had 880 samples processed in 44 plates. Details of the number of cultivars, number of samples, and plates for the laboratory phase of the trial designs are shown in Table 1.

### Statistical analysis

The aim of the analysis is to partition the variation in the sugar release data in each trial into known sources of genetic and non-genetic variation. The non-genetic sources of variation in this experiment are due to the field, laboratory, and trial. First, the factors forming the non-genetic sources of information from the experimental design elements of the field and laboratory phases within each of the trials are discussed. Subsequently, the additional model terms allowing the data of both trials to be analysed as a single multi-environmental trial are determined.

Brien and Bailey [13] provide guidelines on the formulation of the models of a multi-phase experiment. First, identify the objects involved in the randomisation and then determine the factors associated with each set of objects; these groups of factors are referred to as tiers [12]. Once the factors associated with each of the tiers have been identified then the explicit crossing and nesting relationships between factors within each tier based on the experimental randomisation and inherent relationships can be determined and the model terms can be identified.

In this experiment, within each trial, there is the initial randomisation in the field phase of the cultivars to pots, and the subsequent randomisation in the laboratory phase of the cultivar stem samples to the plate plots (that is, to four consecutive wells in the 96-well plate, Figure 2). The first two tiers of each trial therefore contain the unrandomised factors in the laboratory and field phases (Table 3). The third tier contains the factors that are randomised in the field phase, the cultivar. Note the factors in different tiers are associated by randomisation, whereas those in the same tier are not.

For determining the inherent relationship between factors and therefore model terms for the field phase (tier 2, Table 3) the approach of Gilmour *et al*. [9] is used; where the modelling of environmental spatial trends in field trials including global non-stationary trends such as linear row and linear column effects, extraneous terms due to trial management such as random row or column variation, and local trends due to spatial position in the trial are considered. Given each well of the 96 wells in a plate can be defined by a plate row and column position, the model terms for the laboratory phase (tier 1, Table 3) can be determined in a similar way to the field phase terms.

The need for randomisation-based factors due to the trial design, for example replicate block and days, are also considered.

The final model terms and the breakdown of the relevant sources of variation for field and laboratory are shown in an analysis of variance (ANOVA)-like decomposition in Table 4. The model terms relating to field are preceded by an F and those relating to laboratory by an L. The unrandomised field and laboratory phase model terms are described in Table 3 and are treated as random effects in the model.

Now the treatment of the trial, cultivar, and their interaction ensues. The aim is to model the sugar release of cultivars in each of the trials (that is, the cultivar by trial interaction) so that the best cultivars can be selected. The cultivar by trial interaction term is treated as random as the aim is cultivar selection [29]. A main effect for cultivar is not implicitly fitted here and the trial main effect is treated as fixed. This imposes a structure on the interaction term that corresponds to a genetic correlation between trials. Accordingly, a separate variance term is fitted for each trial and the covariance and hence correlation between trials can be estimated [30]. Cultivar main effects can be obtained by use of a selection index or weighted sum of the means across the two trials. As there is a large number of cultivars that overlap across the two trials, also of interest is cultivars that rank consistently well regardless of trial.

The following mixed model is fitted to the saccharification data from both trials as follows:

(1) $$y=X\tau +Z_{g}g+Z_{u}u+\varepsilon$$

The (*n* × 1) ***y*** vector of sugar release (nmol/mg dry matter) consists of the complete data for laboratory samples from both trials, ***τ*** is a (*p* × 1) vector of fixed terms consisting of an overall mean performance for each of the *p =* 2 trials, with associated design matrix ***X***^(*n* × *p*)^, $g^{\left(\text{mp}\times 1\right)}={\left(g_{1}^{T},g_{2}^{T}\right)}^{T}$ is the vector of random cultivar effects of the *m* cultivars in each of the *p* = 2 trials, the variance of var(***g***) = ***G***_*e*_ ⊗ ***I***_*m*_ where ***G***_*e*_ is genetic variance matrix across trials and ***I***_*m*_ the identity matrix, is the genetic variance matrix for cultivars, and ⊗ is the Kronecker product. For two trials, the matrix ***G***_*e*_ has diagonal elements that are the genetic variances for the individual trials $\theta_{g_{1}}^{2}$ and $\theta_{g_{2}}^{2}$ and an off-diagonal element $\theta_{g_{1}g_{2}}$ that is the genetic covariance between the two trials. The off-diagonal element can be used to obtain the genetic correlation between trials.

The vector ***u***^(*b* × 1)^ consists of subvectors $u_{i}^{\left(b_{i}\times 1\right)}$ where the subvector ***u***_*i*_ corresponds to the *i*th random term. The corresponding design matrix $Z_{u}^{\left(b\times 1\right)}$ is partitioned conformably as $\left[Z_{u_{1}}\ldots Z_{u_{b}}\right]$. The subvectors are assumed to be mutually independent with variance $\theta_{i}^{2}I_{b_{i}}$. The subvectors include the random terms for field and laboratory terms (prefixed by F and L, respectively, in Table 3). The residual vector ***ε*** has variance ${⊕}_{p=1}^{2}R_{p}$ a block diagonal matrix of *p* blocks where $R_{p}=\theta_{p}^{2}I_{n_{p}}$ and *n*_*p*_ is the number of observations in trial *p*.

Thus the cultivar term ***g*** reflects the genetic variation and the fixed ***τ***, random ***u***, and residual ***ε*** terms reflect the design and conduct of the two phases of the trials, and as such provide the underlying structure for non-genetic variation.

Because of the unbalanced nature of the data, estimation of variance parameters is by residual maximum likelihood (REML). Given that the variance parameters are estimated, empirical best linear unbiased predictors (EBLUPs) are obtained for random effects such as cultivar effects. Nested variance models are compared using log likelihood ratio tests. The analysis was performed using ASReml for R [31].

When fitting the model described above, a hierarchical or incremental approach must be taken. First, a baseline model is fitted (Equation 2) referred to as model A. This model excludes the field and laboratory terms (terms indexed with an L or F in Table 4) and assumes that the genetic covariance between the two trials $\theta_{g_{1}g_{2}}$ is zero. This is equivalent to fitting each trial separately.

(2) $$y=X\tau +Z_{g}g+\varepsilon$$

The full model (Equation 1) is then fitted, initially assuming the genetic covariance $\theta_{g_{1}g_{2}}$ (model B) between the two trials is zero (equivalent to fitting each trial separately). Once appropriate field and laboratory terms within each trial are determined, the genetic covariance $\theta_{g_{1}g_{2}}$ between the two trials can be estimated (model C). In general, as most of the field and laboratory terms (Table 4) are design factors they are left in the model even if they are not significant, unless the variance component for this term tends to zero.

The calculation of the generalised heritability in complex linear mixed models is not straight-forward [27]. Here the generalised heritability for each trial is calculated as $1-\frac{a}{2\theta_{\text{gp}}^{2}}$ where *a* is the average pairwise prediction error variance of cultivar effects and $\theta_{\text{gp}}^{2}$ is the genetic variance of trial *p*[27].

## Abbreviations

ANOVA: Analysis of variance; DM: Dry matter; EBLUP: Empirical best linear unbiased predictor; FPU: Filter paper unit; GWAS: Genome-wide association study; MBTH: 3-methyl-2-benzothiazolinone hydrazone; MET: multi-environment trial; QTL: Quantitative trait loci; REML: Residual maximum likelihood; UV: Ultraviolet.

## Competing interests

The authors declare that they have no competing interests.

## Authors’ contributions

HO designed the field phase in 2011, designed the laboratory phases in 2010 and 2011, developed the statistic models and conducted the statistical analysis, interpreted the data, and drafted the manuscript. RS supervised and led sample collection and preparation, liaised with the University of York on saccharification, and helped with processing of the data. JC designed the field phase in 2010, assembled the plant material, and coordinated sowing, growing, and sampling for both the 2010 and 2011 trials. NU assisted in setting up the field experiment and sowing, growing, and sampling of plant material for the 2010 and 2011 trials. BC assisted with the development of the statistical model and analysis. LDG set up the high-throughput saccharification platform and supervised the saccharification analysis. CW performed the saccharification analysis. SMM obtained funding for the research and supervised the saccharification analysis. RW conceived the research, obtained funding for the research, supervised the research, and drafted the manuscript. CH conceived the research, obtained funding for and supervised the research, and drafted the manuscript. All authors read and approved the final manuscript.
